# Umbilical artery bypass for surgical treatment of femoral artery stenosis in a newborn: A case report

**DOI:** 10.1016/j.jvscit.2026.102404

**Published:** 2026-07-04

**Authors:** Flora Jin, Alberto Jarrin Lopez, Sunghoon Kim, James Betts

**Affiliations:** a Division of Pediatric Surgery, UCSF Benioff Children's Hospitals, San Francisco, CA; b Department of Surgery, University of California San Francisco-East Bay, Oakland, CA

**Keywords:** Femoral artery stenosis, Newborn, Umbilical artery bypass, Vascular injury

## Abstract

Femoral artery stenosis is a rare but serious complication of arterial puncture in neonates, and surgical revascularization is challenging due to small vessel size. We report the case of a premature newborn with limb-threatening ischemia from femoral artery stenosis treated using the native umbilical artery as an autologous bypass graft. The umbilical artery was mobilized, tunneled, and anastomosed distal to the stenosis, restoring perfusion. Postoperative Doppler confirmed graft patency and resolution of ischemia. The patient later died from unrelated respiratory complications. This case highlights the technical feasibility of umbilical artery bypass for neonatal limb salvage and underscores the vulnerability of premature infants.

Femoral artery stenosis in neonates is a rare but significant complication of arterial puncture injuries during vascular access procedures, often resulting in limb ischemia.^1^ Timely surgical intervention is crucial for restoring blood flow, but traditional options are challenging due to newborns' small size and delicate vascular anatomy. Ultrasound-based measurements demonstrate that neonatal femoral arteries and veins measure only a few millimeters in diameter and vary with gestational age and weight, making standard vascular repair technically difficult.^1^, ^2^, ^3^ In addition, anatomic considerations, such as moving the relative positions of the femoral artery and vein in young children, increase the risk of procedural complications and complicate surgical exposure.^3^

Conventional management for neonatal femoral artery thrombosis relies heavily on anticoagulation therapy, using unfractionated or low-molecular-weight heparin as first-line treatment and reserving operative intervention for cases with persistent ischemia or failure of medical management.^4^ Many acute iliofemoral thrombus resolve medically, although some infants require escalation of therapy or later develop complications when medical treatment is insufficient.^5^ A systematic review highlights the lack of a clear standard of care, particularly for fixed stenotic lesions, reporting substantial variation in practice and limited evidence supporting thrombolysis or surgery in the smallest infants.^6^ As a result, management must be individualized when stenosis is structural rather than thrombotic. Herein, we present a novel approach using the umbilical artery as a bypass graft to treat femoral artery stenosis and restore perfusion to the affected limb. Written informed consent for publication was obtained from the patient's legal guardian, and all identifying information was removed to protect patient confidentiality.

## Case report

A 3-week-old, born at 28 weeks’ gestation, 1.5 kg premature neonate with underlying respiratory distress syndrome in a neonatal intensive care unit developed acute limb-threatening ischemia of the right lower extremity. The ischemia followed a percutaneous femoral artery puncture performed for arterial blood gas analysis. Immediately following the procedure, the limb appeared dusky and cool to the touch. Physical examination and bedside Doppler ultrasound study confirmed the absence of distal pulses, suggesting severe iatrogenic femoral artery stenosis or occlusion. Due to the infant's extreme prematurity and hemodynamic fragility, conventional angiography was deemed unfeasible.

Given the critical nature of the ischemia, the patient was transitioned to the operating room for limb salvage. Upon exploration, the femoral artery was found to be severely stenotic. To restore perfusion without the use of a synthetic graft, the surgical team opted for an umbilical artery bypass. The umbilical artery was mobilized and dissected from its umbilical attachment. The artery was transposed to bypass the stenotic region of the femoral artery (Fig). Flushing of the umbilical artery lumen showed that it was patent, and arterial blood flow was intact. Using microsurgical technique and fine interrupted 9-0 Prolene sutures, a distal anastomosis was performed between the umbilical artery and the patent distal femoral artery. This effectively created a physiological conduit from the iliac system directly to the distal vasculature of the lower extremity (Fig). Intraoperative and postoperative Doppler ultrasound studies demonstrated robust triphasic signals and immediate restoration of capillary refill in the right foot. The patient's postoperative course was stable, and the limb remained well-perfused.

**Fig:**
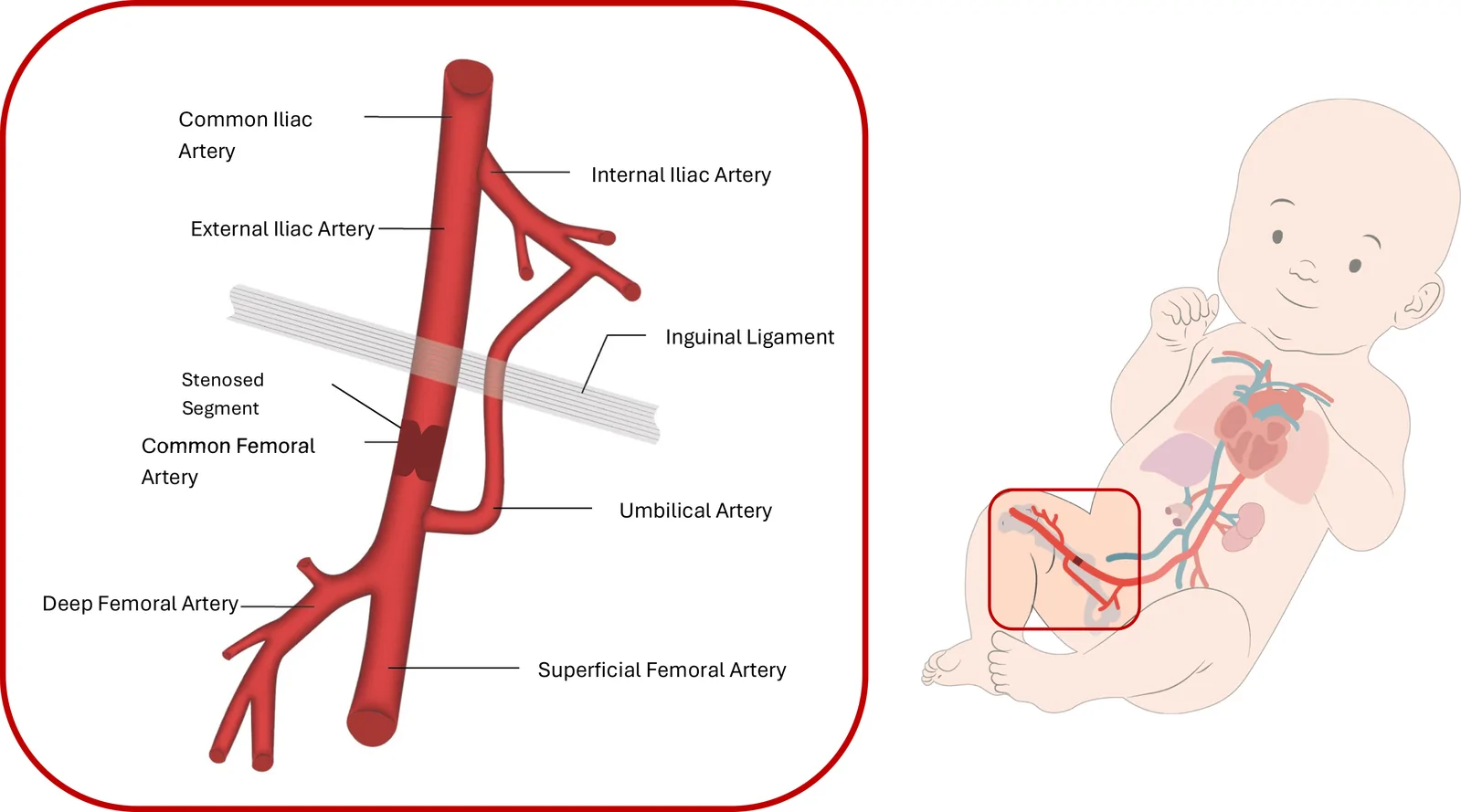
Schematic illustration of the surgical anatomy after anastomosis. The umbilical artery is mobilized and sutured distal to the femoral artery stenosis.

## Discussion

The management of acute limb ischemia in the extremely low-birth-weight neonate represents a formidable surgical challenge. Traditional revascularization options, such as synthetic polytetrafluoroethylene grafts or saphenous vein autografts, are often precluded by the diminutive size of the target vessels and the high risk of thrombotic occlusion. In this case, we successfully used a native umbilical artery autograft as a biological conduit for femoral artery reconstruction, demonstrating its viability for immediate limb salvage.

The umbilical artery is an ideal candidate for neonatal vascular reconstruction for several reasons. Its luminal diameter closely approximates that of neonatal peripheral arteries, facilitating a precise microvascular anastomosis and minimizing hemodynamic turbulence. Originating from the internal iliac artery, its location allows for transposition to the femoral region with minimal additional dissection. Although recent literature focuses on decellularized or vitrified umbilical scaffolds for tissue engineering, these studies confirm that the umbilical artery possesses a robust extracellular matrix and favorable mechanical properties for small-diameter bypass applications.^7^, ^8^, ^9^ The use of a native autograft—rather than a processed scaffold—offers the theoretical advantage of endothelial preservation, which may reduce early graft failure. Although our patient's clinical course was complicated by severe respiratory distress syndrome and subsequent pulmonary failure and death unrelated to the vascular procedure, the intraoperative restoration of distal perfusion and postoperative Doppler patency validate the technical feasibility of this approach.

This case contributes to the limited body of evidence regarding neonatal limb salvage. Although our results are promising, the long-term outcomes regarding graft growth potential and late-term patency remain unknown. Future studies are warranted to compare umbilical artery bypass with alternative techniques, such as microsurgical patch angioplasty, to establish a standardized protocol for iatrogenic neonatal arterial injuries.

## Conclusions

This case demonstrates that the umbilical artery can be successfully used as a bypass conduit for emergent lower-extremity revascularization in very low-birth-weight neonates. Its natural anatomical proximity to the femoral system and its diameter-matching characteristics make it a superior alternative to synthetic materials or smaller venous grafts in this population.

## Funding

None.

## Disclosures

The authors received no financial support for the research, authorship, and/or publication of this article. They have no competing interests.
